# *In silico* and *in vivo* analyses reveal key metabolic pathways enabling the fermentative utilization of glycerol in *Escherichia coli*

**DOI:** 10.1111/1751-7915.13938

**Published:** 2021-10-26

**Authors:** James M. Clomburg, Angela Cintolesi, Ramon Gonzalez

**Affiliations:** ^1^ Department of Chemical and Biomolecular Engineering Rice University Houston TX USA; ^2^ Department of Chemical, Biological, and Materials Engineering University of South Florida Tampa FL USA

## Abstract

Most microorganisms can metabolize glycerol when external electron acceptors are available (i.e. under respiratory conditions). However, few can do so under fermentative conditions owing to the unique redox constraints imposed by the high degree of reduction of glycerol. Here, we utilize *in silico* analysis combined with *in vivo* genetic and biochemical approaches to investigate the fermentative metabolism of glycerol in *Escherichia coli*. We found that *E. coli* can achieve redox balance at alkaline pH by reducing protons to H_2_, complementing the previously reported role of 1,2‐propanediol synthesis under acidic conditions. In this new redox balancing mode, H_2_ evolution is coupled to a respiratory glycerol dissimilation pathway composed of glycerol kinase (GK) and glycerol‐3‐phosphate (G3P) dehydrogenase (G3PDH). GK activates glycerol to G3P, which is further oxidized by G3PDH to generate reduced quinones that drive hydrogenase‐dependent H_2_ evolution. Despite the importance of the GK‐G3PDH route under alkaline conditions, we found that the NADH‐generating glycerol dissimilation pathway via glycerol dehydrogenase (GldA) and phosphoenolpyruvate (PEP)‐dependent dihydroxyacetone kinase (DHAK) was essential under both alkaline and acidic conditions. We assessed system‐wide metabolic impacts of the constraints imposed by the PEP dependency of the GldA‐DHAK route. This included the identification of enzymes and pathways that were not previously known to be involved in glycerol metabolisms such as PEP carboxykinase, PEP synthetase, multiple fructose‐1,6‐bisphosphatases and the fructose phosphate bypass.

## Graphical abstract

Hydrogen evolution and 1,2‐PDO synthesis contribute to redox balancing during fermentative metabolism of glycerol at alkaline and acidic conditions, respectively. Involvement of different glycerol dissimilation pathways depends on the mode of redox balancing. PEP carboxykinase, PEP synthetase, multiple fructose‐1,6‐bisphosphatases, and the fructose phosphate bypass allow the cells to cope with the stoichiometric constraints imposed by the PEP‐dependency of the essential glycerol utilization pathway, GldA‐DhaKLM.

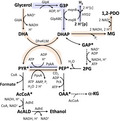

## Introduction

Fermentative and respiratory metabolisms represent the primary modes of cellular energy metabolism. In the absence of external electron acceptors, fermentative metabolism operates utilizing internal electron acceptors to maintain overall redox balance and substrate‐level phosphorylation for ATP generation (Bettenbrock *et al*., 2014). If an external electron acceptor (e.g. O_2_) is available, respiratory metabolism can utilize the electron transport chain and oxidative phosphorylation for redox balancing and ATP generation (Lengeler *et al*., 1999; Nicholls and Ferguson, 2013). These contrasting metabolic modes often rely on different enzymes, pathways and regulatory processes, which has significant consequences on the ability of and mechanisms for microorganisms to utilize carbon and energy source(s) under varying conditions (Bettenbrock *et al*., 2014). For example, although many microorganisms can metabolize glycerol under respiratory conditions, few can do so under fermentative conditions (Clomburg and Gonzalez, 2013).

**Fig. 1:**
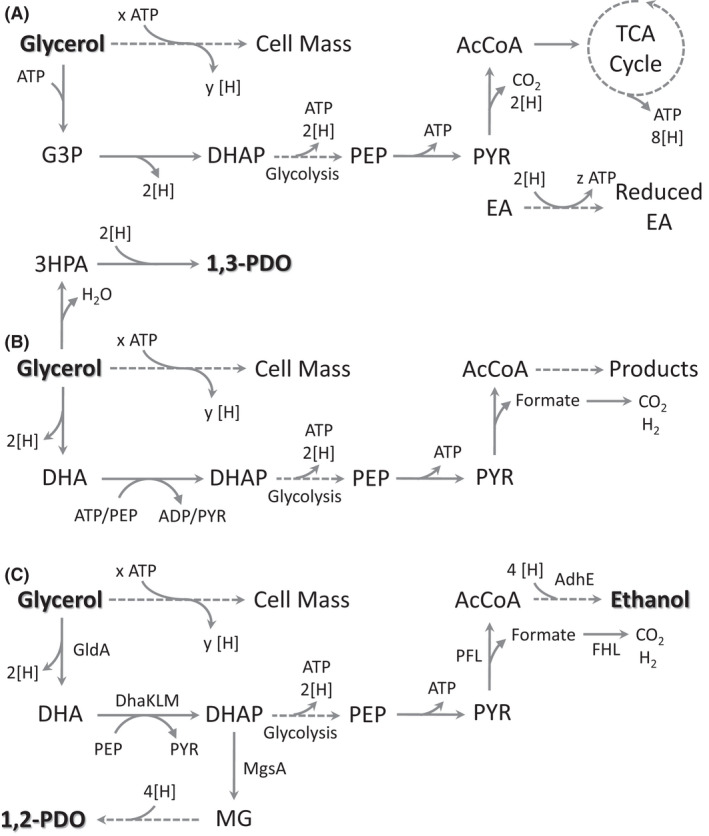
Established metabolic models for microbial glycerol utilization. The highly reduced nature of glycerol results in the generation of excess reducing equivalents during cell mass synthesis that must be consumed to enable redox balance. Models enabling growth on glycerol include the following: (A) respiratory metabolism utilizing electron transfer to an external acceptor and fermentative metabolism involving (B) 1,3‐Propanediol (1,3‐PDO)‐dependent or (C) 1,2‐Propanediol (1,2‐PDO)‐ethanol‐dependent glycerol fermentation. *Escherichia coli* enzymes previously shown to play an important role in fermentative glycerol utilization are shown in (C). Broken lines indicate multiple reaction steps. Broken lines indicate multiple reaction steps. Abbreviations: 1,2‐PDO, 1,2‐propanediol; 1,3‐PDO, 1,3‐propanediol; 2[H] = NADH/NADPH/FADH2; 3HPA, 3‐hydroxypropionaldehyde; AcCoA, acetyl‐CoA; AdhE, alcohol dehydrogenase; DHA, dihydroxyacetone; DhaKLM, PEP‐dependent DHA kinase; DHAP, DHA phosphate; EA, electron acceptor; FHL, formate hydrogen lyase; G3P, glycerol‐3‐phosphate; GldA, glycerol dehydrogenase; MG, methylglyoxal; MgsA, methylglyoxal synthase; PEP, phosphoenolpyruvate; PFL, pyruvate formate lyase; PYR, pyruvate; TCA, tricarboxylic acid

A critical factor in the dichotomy between fermentative and respiratory glycerol utilization is the highly reduced nature of this carbon source (κ = 4.67; degree of reduction per carbon, κ, a measure of the number of available electrons per unit of carbon). With more oxidized carbon sources such as glucose (κ = 4), the synthesis of cell mass (κ = 4.3, based on average biomass molecular formula of CH_1.9_O_0.5_N_0.2_) results in a net consumption of reducing equivalents (Nielsen *et al*., 2003). However, the synthesis of cell mass from (i.e. growth on) glycerol generates reducing equivalents, tying a microorganism's ability to maintain redox poise to a sink for these excess reducing equivalents (Clomburg and Gonzalez, 2013). Under respiratory conditions, this is achieved via electron transfer to an external acceptor (Fig. 1A); however, options are much more limited under fermentative conditions where redox balance must be maintained by regenerating oxidized electron carriers using internal metabolites. The most well‐established means of enabling fermentative glycerol utilization are through the synthesis of highly reduced 1,3‐propanediol (1,3‐PDO, κ = 5.33), a model extensively studied in several species of the *Enterobacteriaceae* family (Fig. 1B; Bouvet *et al*., 1995; Sun *et al*., 2003). While the absence of 1,3‐PDO‐producing capability was long thought to confine microorganisms to respiratory glycerol metabolism, fermentative glycerol utilization has been reported in non‐1,3‐PDO‐producing organisms such as *Escherichia coli* (Dharmadi *et al*., 2006; Zhang *et al*., 2010), *Paenibacillus macerans* (Gupta *et al*., 2009) and *Propionibacterium acidipropionici* (Bories *et al*., 2004).

Fermentative glycerol utilization by *E. coli* has been further established and linked to its ability to produce 1,2‐propanediol (1,2‐PDO, κ = 5.33), whose synthesis from glycerol results in the net consumption of NADH, although it also consumes ATP (Fig. 1C; Gonzalez *et al*., 2008). Ethanol synthesis was also shown to play an essential role as the overall glycerol to ethanol conversion is redox‐balanced and enables the synthesis of ATP through glycolysis (Fig. 1C; Gonzalez *et al*., 2008). Another key feature of this ethanol‐1,2‐PDO metabolic model is the conversion of glycerol to the glycolytic intermediate dihydroxyacetone phosphate (DHAP), a key node for both 1,2‐PDO and ethanol synthesis, through a type II glycerol dehydrogenase (GldA) and phosphoenolpyruvate (PEP)‐dependent dihydroxyacetone (DHA) kinase (DhaKLM; Fig. 1C). While the identification of these key pathways has facilitated metabolic engineering efforts aimed at the production of numerous value‐added products (Blankschien *et al*., 2010; Mazumdar *et al*., 2010; Clomburg and Gonzalez, 2011; Cintolesi *et al*., 2012), glycerol utilization under these conditions is still poorly understood.

Here, we utilize a genome‐scale model (GEM) of *E. coli* in combination with Flux Balance Analysis (FBA) and Flux Variability Analysis (FVA) to investigate the distribution of metabolic fluxes during the fermentative utilization of glycerol in *E. coli*. This *in silico* analysis facilitated the identification of key areas within *E. coli* metabolism that was further investigated *in vivo* using genetic and biochemical approaches. Our findings reveal that *E. coli* possesses multiple means for achieving redox balance during fermentative glycerol utilization dependent on environmental conditions such as pH, with proton reduction to hydrogen (H_2_) via a reversible hydrogenase critical under alkaline conditions. H_2_‐dependent redox balancing can improve ATP generation compared with 1,2‐PDO production previously shown to operate under acidic conditions and is enabled by the activity of the respiratory glycerol dissimilation pathway which generates electrons in a form amenable to hydrogenase‐mediated proton reduction. However, owing to the pH dependence of proton reduction via hydrogenases and the inability of the respiratory pathway to exclusively support glycerol fermentation, the previously established GldA‐DhaKLM dissimilation route was found to be essential under both alkaline and acidic conditions. The stoichiometric coupling imposed by the PEP dependence of DHA kinase further underlines the importance of several enzymes and pathways that were not previously known to be involved in glycerol metabolism. Together, these findings demonstrate how the robust metabolic network of *E. coli* manages the stoichiometric, redox and ATP generation constraints associated with the fermentative metabolism of glycerol.

## Results and discussion

### Hydrogen evolution and 1,2‐PDO synthesis both contribute to redox balance during fermentative metabolism of glycerol

**Fig. 2:**
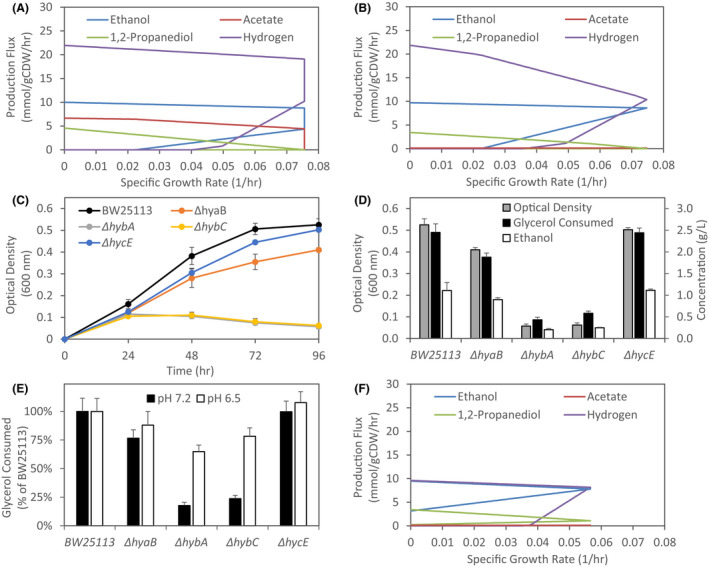
Analysis of key products during glycerol fermentation in *E. coli*. *in silico* modelling of predicted product solution spaces for: (A) initial curated *E. coli* genome‐scale model (GEM), (B) base case in which acetate and succinate production were fixed to previously observed experimental values and reactions enabling the transfer of electrons from NAD(P)H to the quinone pool were removed (see text), and (F) base case upon removal of the reversible menaquinol‐coupled hydrogenase reaction. This analysis identified key enzymes involved during glycerol fermentation that were further examined through the evaluation of specific gene deletions on the (C) cell growth profile and (D) glycerol consumption, ethanol synthesis and cell growth after 96 h under alkaline conditions (pH 7.2). *Escherichia coli* K12 strain BW25113 was used as our parental strain for all experiments. Number of independent biological replicates: BW25113, *n* = 45; Δ*hyaB*, *n* = 4; Δ*hybA*, *n* = 4; Δ*hybC*, *n* = 4; and Δ*hycE*, *n* = 4. The impact of individual deletion of genes encoding essential hydrogenase 1, 2 and 3 subunits on glycerol consumption under acidic conditions (pH 6.5) is also shown in comparison with alkaline conditions (E). Glycerol consumption is shown as a percentage consumption by the parent strain BW25113 under the indicated conditions. Number of independent biological replicates for pH 6.5: BW25113, *n* = 4; Δ*hyaB*, *n* = 2; Δ*hybA*, *n* = 2; Δ*hybC*, *n* = 2; and Δ*hycE*, *n* = 2.

Utilizing a curated genome‐scale model for *E. coli* (GEM: see Appendix S1 for details), Flux Balance Analysis (FBA) and Flux Variability Analysis (FVA) were used to predict cell growth and flux distributions and solution spaces, respectively (Fang *et al*., 2020). Optimization to maximize biomass resulted in a specific growth rate of 0.075 h^−1^, with product solution spaces revealing the existence of multiple solutions with varying product fluxes (i.e. the edge of the curve defining the maximum specific growth in Fig. 2A). Ethanol and H_2_ represent growth‐coupled products, with all optimal solutions for biomass maximization requiring non‐zero fluxes (Fig. 2A). The production of and growth‐coupling to ethanol are expected based on the redox‐balanced and ATP‐generating nature of the pathway from glycerol to ethanol and formate (or glycerol to ethanol, CO_2_ and H_2_: C_3_H_8_O_3_ → C_2_H_6_O + CH_2_O_2_/CO_2_‐H_2_). However, the prediction of significant acetate production and non‐essentiality of 1,2‐PDO synthesis as part of optimal solution(s) maximizing biomass formation (Fig. 2A) represents a phenotype not previously observed experimentally. To account for this deviation, acetate production was constrained in the model to a previously observed molar yield (0.012 mol mol^−1^ glycerol consumed; Murarka *et al*., 2008). Based on these previous experiments, succinate production was also constrained (0.015 mol mol^−1^ glycerol consumed). Furthermore, reactions enabling the transfer of electrons from NAD(P)H to the quinone pool were removed, as the expression of enzymes responsible for this conversion (NADH:ubiquinone oxidoreductase I and II) is induced by O_2_ or other external electron acceptors (Bongaerts *et al*., 1995; Tran *et al*., 1997). Simulations with these constraints indicated that cycling between malate/fumarate and glycolate/glyoxylate reactions was occurring to shift electrons between NAD(P)H and quinones, which do not represent a phenotype experimentally observed, and hence, these cycles were removed as well.

With this updated model, optimization to maximize biomass resulted in single optima for ethanol and H_2_ production at the maximum specific growth rate of 0.075 h^−1^, with 1,2‐PDO production again a non‐essential product (Fig. 2B). H_2_ production is higher than that of ethanol (plus acetate; Fig. 2B), indicating that its generation is not solely from formate oxidation. It should be noted that conversion of pyruvate to acetyl‐CoA was predicted to take place exclusively via pyruvate formate lyase (PFL) with no flux through pyruvate dehydrogenase (PDH). While PDH is active during the fermentation of more oxidized carbon sources such as glucose (κ_glucose_ = 4; Murarka *et al*., 2010b) and glucuronate (κ_glucuronic acid_ = 3.33; Murarka *et al*., 2010a), negligible activity is expected due to PDH inhibition by NADH under the highly reduced intracellular environment generated by glycerol (κ_glycerol_ = 4.67) fermentation (de Graef *et al*., 1999; Sawers and Clark, 2004).

Given the role of H_2_ evolution via hydrogenases (HYDs) predicted by the *in silico* analysis, we investigated the impact of gene deletions impairing hydrogen metabolism (genes encoding subunits of various hydrogenases). In addition to specific gene deletions, an important consideration when evaluating the role of hydrogen metabolism is the pH at which fermentations are conducted. Our previous studies have demonstrated that disruption of HYD3, that together with formate dehydrogenase‐H (FdhF, encoded by *fdhF*) forms the membrane‐anchored formate hydrogen lyase (FHL) system (Sargent, 2016), has a negative impact on glycerol fermentation under acidic pH, but negligible impact under alkaline conditions (Dharmadi *et al*., 2006; Gonzalez *et al*., 2008; Murarka *et al*., 2008). Furthermore, reports have demonstrated that overall HYD activity and the involvement of specific HYDs in H_2_ production by *E. coli* cells grown in the presence of glycerol are pH dependent, with hydrogenase 1 (HYD1) and 2 (HYD2) involved at alkaline pH and hydrogenase 3 (HYD3) responsible for H_2_ production at acidic pHs (Trchounian and Trchounian, 2009; Trchounian *et al*., 2012; Trchounian *et al*., 2013). Based on these previous findings and the model prediction of H_2_ generation not solely from formate oxidation, we initially conducted fermentations at an alkaline pH (7.2). To facilitate the investigation into numerous gene deletions, we utilized the parent strain *E. coli* BW25113 and associated single‐gene deletion mutants of the Keio collection (Baba *et al*., 2006). For hydrogenases, these included strains with deletions documented to impair hydrogenase 1, 2 and 3: *hyaB*, encoding the large subunit of HYD1 that contains the [NiFe] active site (Menon *et al*., 1991), *hybA* encoding the iron–sulphur protein shown to be essential for reversible electron transfer between HYD2 and the quinone pool (Pinske *et al*., 2015), *hybC* encoding the large, [NiFe]‐containing subunit of HYD2 (Dubini *et al*., 2002) and *hycE* encoding the large subunit of HYD3 (Sauter *et al*., 1992). Cell growth and glycerol consumption were evaluated for fermentations conducted under strict anaerobic conditions (see *Experimental procedures*) with time profiles assessed in 24‐h intervals based on our previous demonstration of exponential growth extending through at least the first 30 h of cultivation (Murarka *et al*., 2008). As glycerol fermentation at both acidic and alkaline pH is homoethanologenic in nature (Murarka *et al*., 2008), ethanol synthesis was also utilized as a key metric to evaluate the impact of various gene deletions.

Under these conditions, fermentative glycerol utilization was observed in BW25113 with ethanol representing the predominant product as expected (Fig. 2C and D). Interestingly, estimating the average specific growth rate during the first 24 h, assuming a short‐lag phase, results in a value comparable with the model prediction (~0.08 experimentally vs 0.075 h^−1^ from the model), which are both higher than the previously reported growth rates under acidic conditions (0.04 h^−1^; Murarka *et al*., 2008) where 1,2‐PDO production was identified during glycerol fermentation. Analysis of gene deletions impairing hydrogen metabolism also revealed that while HYD3 disruption has a minimal impact under these alkaline conditions, aligning with previous findings, disruption of HYD1 (Δ*hyaB*) and especially HYD2 (Δ*hybA*, Δ*hybC*) resulted in reduction to cell growth, glycerol consumption and ethanol synthesis (Fig. 2C and D). While no significant difference in growth or glycerol consumption was observed in the Δ*hycE* strain, there was a near fourfold increase in formate concentration compared with BW25113 (1.13 ± 0.04 vs 0.31 ± 0.02 g L^−1^). Similar high formate production and minimal impact to cell growth and glycerol consumption were observed in a Δ*fdhF* strain (data not shown), indicating that *hycE* deletion resulted in a non‐functional FHL complex. To further assess the impact of individual hydrogenase disruption in the context of pH, we evaluated BW25113 and hydrogenase deletion strains at an acidic pH (6.5). As shown in Fig. 2E, under these conditions disruption of the various hydrogenases had a much less pronounced impact on glycerol utilization compared with alkaline pH, with only Δ*hybA* and Δ*hybC* strains deficient in HYD2 resulting in slight decreases to glycerol consumption levels compared with BW25113. It should be noted that the minimal impact of HYD3 disruption under acidic conditions here is likely the result of CO_2_ included in the headspace, as our previous results have demonstrated the negative impact of FHL disruption on glycerol fermentation under acidic conditions is mitigated by a partial CO_2_ headspace atmosphere (Gonzalez *et al*., 2008).

The above results clearly demonstrate a role of hydrogenases, specifically HYD1 and HYD2, during the fermentative utilization of glycerol that is dependent on external pH. While HYD1 and HYD2 have commonly been thought to act as H_2_‐uptake enzymes, both enzyme complexes have been shown to contribute to H_2_ production depending on the external pH and carbon source, including cells grown in the presence of glycerol (Trchounian and Trchounian, 2009; Trchounian *et al*., 2013, 2017). Furthermore, the O_2_‐sensitive HYD2 is well adapted to reduced environments to the extent that it can act as an electron release valve if the quinone pool becomes over‐reduced when electron acceptors are limited (Lukey *et al*., 2010). This behaviour, combined with the model prediction of the importance of H_2_ production and our experimental validation that glycerol fermentation significantly benefits from functional HYD1 and HYD2 complexes at alkaline pH, supports a scenario in which the highly reduced nature of glycerol and absence of external electron acceptors result in proton reduction to H_2_ serving as a mechanism for redox balancing during glycerol fermentation under certain conditions. Importantly, the ability for HYD2 to catalyse H_2_ evolution has been shown to occur with glycerol as the carbon source using electrons in the form of menaquinol or demethylmenaquinol (Pinske *et al*., 2015). Furthermore, the reversibility of HYDs, including that of HYD2, has been shown to rely on the proton motive force (PMF; Trchounian *et al*., 2013; Pinske *et al*., 2015), while the activity of HYDs both depends on and contributes to F_0_F_1_‐ATPase activity (Trchounian *et al*., 2011, 2013). During glycerol fermentation, it has been proposed that coupling of HYD and proton ATPase activities to regulate intracellular pH contributes to overall PMF generation, which is pH dependent (Trchounian and Trchounian, 2019). Thus, while HYD‐dependent proton reduction may enable H_2_ production to consume excess reducing equivalents, the reliance on the PMF may dictate pH dependence. Our findings of the importance of HYDs here support this proposed involvement and suggest proton reduction to H_2_ can serve as a sink for excess reducing equivalents generated from biomass formation during glycerol fermentation at alkaline pH. An alternative mechanism such as 1,2‐PDO production is required under conditions in which HYD reversibility is less favourable (e.g. acidic conditions).

To compare 1,2‐PDO synthesis and HYD‐dependent H_2_ production as enablers of glycerol fermentation under acidic or alkaline conditions, respectively, we used *in silico* modelling to determine the solution space when the reversible HYD reaction involving menaquinol was removed from the reaction network. In this case, optimization to maximize biomass formation resulted in a lower specific growth rate (0.057 h^−1^), with ethanol remaining the predominant product and 1,2‐PDO now representing a growth‐coupled product (Fig. 2F), findings that align with both previously determined growth rates and the importance of these products at acidic pH (Dharmadi *et al*., 2006; Gonzalez *et al*., 2008; Murarka *et al*., 2008). While H_2_ remained growth‐coupled, its production was at stoichiometric levels to ethanol (plus acetate) indicating its generation solely from formate oxidation via FHL (Fig. 2F). These findings demonstrate that *E. coli* can use either 1,2‐PDO or HYD‐dependent H_2_ production as a sink for excess reducing equivalents, thus achieving redox balance. While the H_2_ evolution route appears more efficient and results in higher specific growth rates, the pH dependence of this mechanism limits the conditions under which it plays a prominent role. This higher efficiency could relate to energy demands of 1,2‐PDO production, as its synthesis from glycerol results in the net consumption of an ATP molecule (Fig. 1C). Furthermore, in contrast to diverting a fraction of carbon to 1,2‐PDO solely to consume reducing equivalents, H_2_ evolution also enables increased carbon flux to ethanol synthesis with the corresponding increase in substrate‐level phosphorylation via glycolysis resulting in further improved ATP yield.

### Involvement of different glycerol dissimilation pathways depends on the mode of redox balancing

**Fig. 3:**
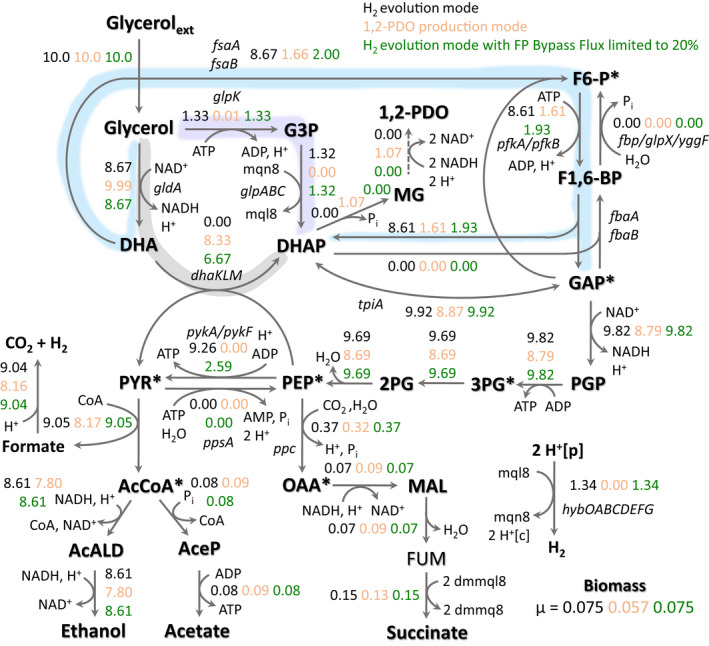
Predicted flux distributions during the fermentative metabolism of glycerol by *E. coli* using the H_2_ evolution mode corresponding to the GEM utilized in Fig. 2B (black text), 1,2‐PDO production mode in which the reversible menaquinol‐coupled hydrogenase reaction was removed from the GEM (orange text), and the H_2_ evolution mode in which flux through the fructose phosphate (FP) bypass was constrained to a maximum of 20% of the incoming glycerol transport flux (green text). Various glycerol dissimilation pathways are highlighted by shaded areas and included the GldA‐DhaKLM‐mediated pathway (grey), GlpK‐GlpABC‐mediated pathway (light purple) and the FP bypass (light blue). Abbreviations: 1,2‐PDO, 1,2‐propanediol; 2 dmmq8, 2‐demethylmenaquinone 8; 2 dmmql8, 2‐demethylmenaquinol 8; 2PG, 3‐phosphoglycerate; 3PG, 3‐phosphoglycerate; AcALD, acetaldehyde; AcCoA, acetyl‐CoA; AceP, acetyl phosphate; CoA, Coenzyme A; DHA, dihydroxyacetone; DHAP, DHA phosphate; F1,6‐BP, fructose‐1,6‐bisphosphate; F6‐P, fructose‐6‐phosphate; FUM, fumarate; G3P, glycerol‐3‐phosphate; GAP, glyceraldehyde‐3‐phosphate; MAL, malate; MG, methylglyoxal; mql8, menaquinol 8; mqn8, menaquinone 8; OAA, oxaloacetate; PEP, phosphoenolpyruvate; PGP, 3‐phosphoglyceroyl phosphate; PYR, pyruvate. The asterisk denotes precursor metabolite required for cell biosynthesis.

An interesting facet to the role of H_2_ evolution and hydrogenase activity in overall redox balance is the form of electrons donor(s) that can facilitate proton reduction to H_2_. As HYD1 and HYD2 operate in conjunction with the quinone pool, their involvement during fermentative glycerol metabolism implies the generation of reduced quinone (e.g. menaquinol) to enable proton reduction. However, previous studies on fermentative glycerol metabolism under 1,2‐PDO‐dependent, acidic conditions have demonstrated the essentiality of an NADH‐generating glycerol oxidation pathway mediated by GldA and DhaKLM under these conditions (Dharmadi *et al*., 2006; Gonzalez *et al*., 2008; Murarka *et al*., 2008). To investigate how glycerol dissimilation, central carbon metabolism, hydrogen metabolism and overall redox balance are connected under various conditions, we examined *in silico* flux distributions for the scenarios of alkaline H_2_‐enabled (Fig. 3, black text) and acidic 1,2‐PDO‐enabled (Fig. 3, orange text) glycerol fermentation. In addition to the NADH‐generating GldA and DhaKLM‐mediated pathway (Fig. 3, shaded grey), for the H_2_‐enabled scenario flux through the respiratory pathway composed of glycerol kinase (GlpK) and anaerobic glycerol 3‐phosphate dehydrogenase (GlpABC) was also predicted (Fig. 3, shaded purple). The involvement of the GlpK‐GlpABC pathway is linked to HYD‐mediated H_2_ evolution through the generation of menaquinol from glycerol‐3‐phosphate (G3P) oxidation, demonstrating how operation of a respiratory pathway under anaerobic conditions could result in electron donor(s) facilitating proton reduction (Fig. 3). In addition to these two routes, flux through a dissimilation pathway involving fructose‐1,6‐biphosphate (F1,6‐BP) as an intermediate was also predicted (Fig. 3, shaded light blue). This pathway, herein termed the fructose phosphate (FP) bypass, involves aldol condensation of DHA, the product of glycerol oxidation via GldA and glyceraldehyde 3‐phosphate forming fructose 6‐phosphate (F6‐P), which is subsequently phosphorylated to F1,6‐BP and cleaved to glyceraldehyde 3‐phosphate and DHAP (Fig. 3). While this pathway has not been implicated in glycerol dissimilation to date, the *gldA* operon also includes *fsaB*, encoding a F6‐P aldolase (FsaB). Both FsaB and another F6‐P aldolase, FsaA (encoded by *fsaA*), have been shown to catalyse the aforementioned aldol condensation (Schurmann and Sprenger, 2001).

Given these findings, we conducted FVA to determine the minimum and maximum reaction fluxes that support the maximum predicted biomass flux for the H_2_‐enabled and 1,2‐PDO‐enabled scenarios (0.075 and 0.057 h^−1^, respectively). This analysis showed that glycolysis and product synthesis fluxes could not vary, expected based on the single optima observed in the product solution spaces (Fig. 2B and F); however, a significant amount of flux variability through glycerol dissimilation pathways can be tolerated (Table S1). This involves modulating flux through the NADH‐generating GldA‐DhaKLM and FP bypass pathways, at the expense of one another, as the flux through the menaquinol‐generating GlpK‐GlpABC pathway was fixed at a value equal to HYD‐dependent H_2_ evolution (Table S1). Thus, from a stoichiometric standpoint there is not a discernable difference in either NADH‐generating glycerol dissimilation pathway; however, concurrent operation of NADH‐ and menaquinol‐generating dissimilation pathways is required for HYD‐dependent H_2_ evolution. This reflects a scenario in which coupling G3P oxidation to proton reduction enables H_2_ production to serve as a sink for excess reducing equivalents; however, the GlpK‐GlpABC route is unable to exclusively support glycerol fermentation.

**Fig. 4:**
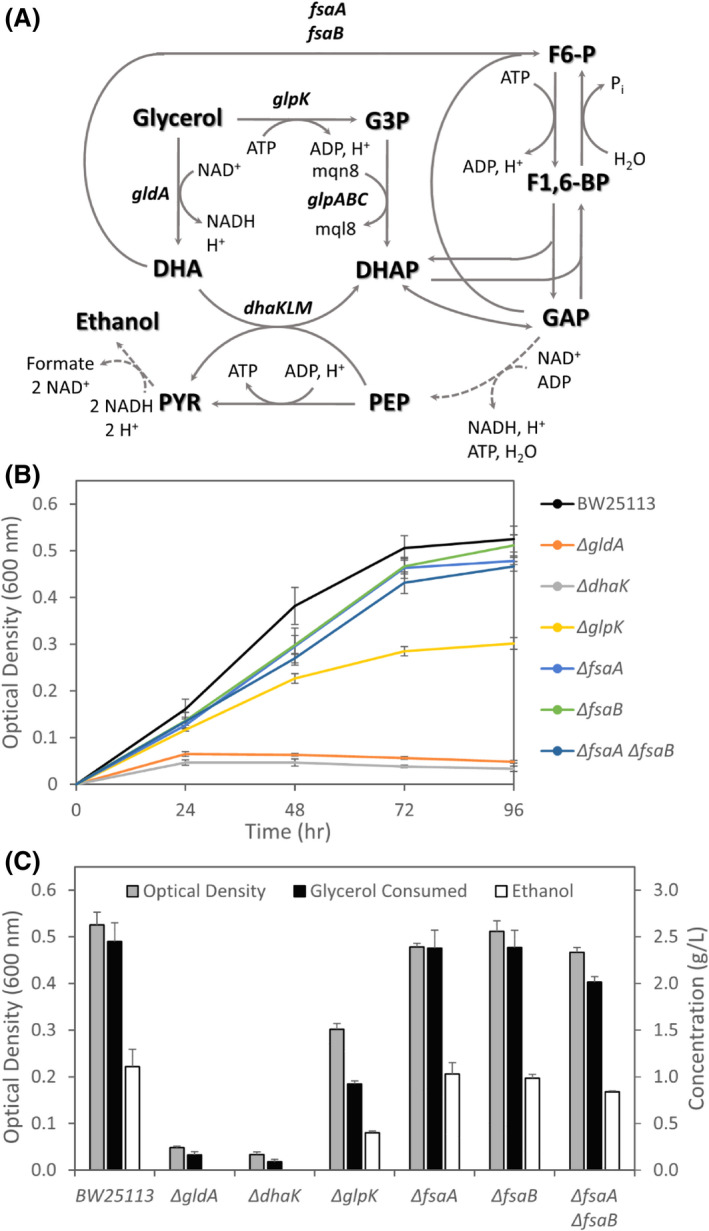
*In vivo* analysis of glycerol dissimilation pathways under fermentative conditions. (A) Potential pathways involved in the conversion of glycerol to glycolytic intermediates. Broken lines indicate multiple reaction steps. Gene(s) encoding‐specific enzymes catalysing each step are listed and the impact of their deletion on the (B) cell growth profile and (C) glycerol consumption, ethanol synthesis and cell growth after 96 h shown. Number of independent biological replicates: BW25113, *n* = 45; Δ*gldA*, *n* = 3; Δ*dhaK*, *n* = 3; Δ*glpK*, *n* = 3; Δ*fsaA*, *n* = 3; Δ*fsaB*, *n* = 3; and Δ*fsaA* Δ*fsaB*, *n* = 3. Abbreviations: α‐KG, α‐ketoglutarate; see Fig. 3 for remaining list of abbreviations.

To determine the importance of these dissimilation pathways *in vivo*, we utilized BW25113 derivatives with deletions to *gldA* (encoding GldA), *dhaK* (encoding a subunit of DhaKLM), *glpK* (encoding GlpK) as well as *fsaA* and *fsaB*, encoding the two distinct F6‐P aldolases (Fig. 4A). Given the lack of knowledge on the role of these F6‐P aldolases, we also constructed a Δ*fsaA* Δ*fsaB* double mutant. Under alkaline conditions, disruption of either GldA or DhaKLM resulted in near abolishment of glycerol consumption (Fig. 4B and C), confirming their essential role during glycerol fermentation. *glpK* deletion also had a significant impact under these conditions, with more than twofold reduction in glycerol consumption observed (Fig. 4B and C). Further confirming the involvement of these dissimilation pathways, activity of GldA, DhaKLM, GlpK and GlpABC was all detected in strain BW25113 while GlpD activity was not (Table S2). However, under acidic conditions the *glpK* deletion had a less pronounced impact (Fig. S1) providing further evidence towards the link between operation of this respiratory pathway and HYD activity and the associated pH dependence.

The combined deletion of both F6‐P aldolases also decreased glycerol consumption (*M* = 2.01 g L^−1^, SD = 0.06 g L^−1^) compared with BW25113 (*t*(5.97) = 9.56, *p* < 0.01; Fig. 4C). Intriguingly, individual deletions to *fsaA* and *fsaB* did not have a significant impact on glycerol consumption, indicating that either enzyme may be able to complement for deletion of the other. It should also be noted that while *gldA* deletion results in disruption of both the FP bypass and the GldA‐DhaKLM pathway, the lack of glycerol consumption in the Δ*dhaK* strain demonstrates the essential nature of the GldA‐DhaKLM route under both acidic and alkaline conditions (Fig. 4C).

Overall, these findings demonstrate the importance of parallel operation of NADH‐ and menaquinol‐generating dissimilation pathways under alkaline conditions in which HYD1 and HYD2 play an important role. Given the potential but non‐essential role of the FP bypass (Fig. 4C), we refined our *in silico* model by limiting the upper bound flux through this pathway to 20% of the incoming glycerol transport flux, a value representative of the decrease in glycerol consumption observed in the Δ*fsaA* Δ*fsaB* strain. Optimization to maximize biomass formation with this refined model results in a specific growth rate of 0.075 h^−1^, with the flux distribution predicting glycerol dissimilation through all 3 routes (Fig. 3, green text). Additional FVA analysis confirmed that FP bypass flux can vary in the permissible range, with associated variability through GldA‐DhaKLM, while flux through GlpK‐GlpABC did not allow for variability at the maximum growth rate (Table S1). These imposed constraints serve to confine glycerol dissimilation fluxes in agreement with our *in vivo* data, allowing us to further investigate perturbations in other areas of the metabolic network.

### Impact of stoichiometric constraints imposed by the PEP dependency of the essential glycerol utilization pathway, GldA‐DhaKLM

**Fig. 5:**
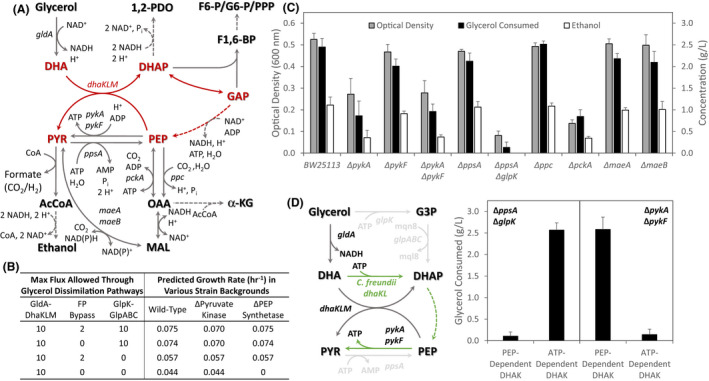
Implications of PEP‐coupled glycerol dissimilation via GldA‐DhaKLM. (A) Metabolic cycle created by the involvement of PEP‐dependent DhaKLM during glycerol dissimilation (shown in red). Gene(s) encoding‐specific enzymes catalysing each step are listed. (B) FBA predicted specific growth rates in response to removing various glycerol dissimilation pathways in combination with key reactions at the PEP node. (C) *In vivo* impact of the deletion of specific genes involved at the PEP node, shown in Panel A, on glycerol consumption, ethanol synthesis, and cell growth after 96 h. Number of independent biological replicates: BW25113, *n* = 45; Δ*pykA*, *n* = 6; Δ*pykF*, *n* = 6; Δ*pykA* Δ*pykF*, *n* = 6; Δ*ppsA*, *n* = 3; Δ*ppsA* Δ*glpK*, *n* = 6; Δ*ppc*, *n* = 6; Δ*pckA*, *n* = 6; Δ*maeA*, *n* = 3; and Δ*maeB*, *n* = 3. (D) Effect of overexpressing fully decoupled (ATP‐dependent DHA kinase from *C. freundii* and *E. coli* GldA) or PEP‐coupled (*E. coli* PEP‐dependent DhaKLM and GldA) glycerol dissimilation pathways *in vivo*. Grey lines indicate reactions/pathways removed in Δ*ppsA* Δ*glpK* strain. Green lines indicate reactions/pathways for DHA to PYR conversion required when using ATP‐dependent DHA kinase. Glycerol consumption after 96 h shown for host backgrounds without either a secondary decoupling mechanism (Δ*ppsA* Δ*glpK*) or pyruvate kinases (Δ*pykA* Δ*pykF*) transformed with pZS plasmid overexpressing the indicated glycerol dissimilation pathway. Number of independent biological replicates: Δ*ppsA* Δ*glpK* (pZSKLMgldA), *n* = 3; Δ*ppsA* Δ*glpK* (pZSKLcfgldA), *n* = 3; Δ*pykA* Δ*pykF* (pZSKLMgldA), *n* = 3; and Δ*pykA* Δ*pykF* (pZSKLcfgldA), *n* = 3. Broken lines indicate multiple reaction steps. Abbreviations: α‐KG, α‐ketoglutarate; G6‐P, glucose‐6‐phosphate; PPP, pentose phosphate pathway; see Fig. 3 for remaining list of abbreviations.

Despite contributions to glycerol dissimilation from three different pathways, both the *in silico* analysis and the experimental evidence clearly established that the GldA‐DhaKLM pathway is the only one essential for glycerol fermentation. This essentiality places unique constraints on glycerol fermentation as DhaKLM uses PEP as the phosphate group donor for DHA phosphorylation. While PEP‐dependent phosphorylation systems exist in nature for other substrates, such as glucose, the fact that both the substrate (glycerol) and the phosphate donor (PEP) are 3‐C molecules imposes distinctive restrictions. Unlike glucose where the phosphorylated product (glucose‐6‐phosphate) is converted into two 3‐C molecules (one of which is used to regenerate the PEP utilized for phosphorylation), for 3‐C glycerol this creates a cycle in the metabolic network: every mole of DHA converted to DHAP is coupled to the conversion of one mole of PEP to pyruvate (Fig. 5A). This stoichiometric constraint greatly limits the availability of PEP and all 3‐C intermediates upstream of PEP and hence the synthesis of biosynthetic precursors or products (e.g. 1,2‐PDO) derived from them.

To probe this coupling, we investigated how removing various glycerol dissimilation pathways from the *in silico* reaction network in combination with key reactions interconverting PEP and pyruvate impacts the predicted growth rate. The latter included the pyruvate kinase reaction, converting PEP to pyruvate with concomitant ATP generation, and PEP synthetase, encoded by *ppsA*, a gluconeogenic enzyme which phosphorylates pyruvate to PEP requiring 2 ATP equivalents (ATP is converted to AMP; Fig. 5A; Niersbach *et al*., 1992). As seen in Fig. 5B, the *in silico* modelling predicts that removal of pyruvate kinase has a slight impact on growth rate when GlpK‐GlpABC is present. However, the removal of the two PEP‐decoupled glycerol dissimilation pathways (i.e. GlpK‐GlpABC and FP bypass) significantly decreased the predicted specific growth rate to 0.044 h^−1^ with no further effect from pyruvate kinase removal. While this lower growth rate hints at the constraints placed on the metabolic network when glycerol dissimilation is fully coupled to PEP, the true implications of this are seen by the prediction that removing PEP synthetase in the absence of a PEP‐decoupled pathway completely inhibits cell growth (Fig. 5B). Thus, without an additional dissimilation pathway that can uncouple glycerol dissimilation from requiring PEP, cells are forced to utilize the ATP intensive PEP synthetase to regenerate PEP from pyruvate to enable growth.

To investigate these predictions, we assessed the impact of deletion of genes encoding enzymes that catalyse the corresponding reactions, including pyruvate kinases I (*pykF*) and II (*pykA*), as well as a Δ*pykF* Δ*pykA* double mutant (Ponce *et al*., 1995). *pykF* deletion had marginal impact on glycerol consumption, while a *pykA* deletion decreased glycerol consumption more than twofold, with the double mutant exhibiting a similar decrease (Fig. 5C). This suggests that conversion of PEP to pyruvate via the kinase reaction is important, with PykA responsible under the examined conditions, implying the operation of a PEP‐independent dissimilation pathway (i.e. GlpK‐GlpABC and FP bypass, as presented in the previous section). To further demonstrate the relationship between a PEP‐independent dissimilation pathway(s) and pyruvate kinase activity, we overexpressed an ATP‐dependent (PEP‐independent) DHA kinase from *Citrobacter freundii* (*Cf*DhaKL; Daniel *et al*., 1995) in conjunction with GldA in the Δ*pykF* Δ*pykA* strain. While minimal glycerol consumption is observed with *Cf*DhaKL overexpression, significant consumption is seen with overexpression of *E. coli* DhaKLM along with GldA in this background (Fig. 5D) showing the importance of pyruvate kinase activity with a PEP‐independent dissimilation pathway.

The importance of a PEP‐independent glycerol dissimilation pathway(s) becomes more evident in a Δ*ppsA* background. Following the *in silico* results, while individual disruption of PEP synthetase had minimal impact *in vivo*, the further deletion of *glpK* resulted in significant decreases in cell growth and glycerol consumption (Fig. 5C). The impact of this Δ*ppsA* Δ*glpK* background is as severe as the disruption of GldA‐DhaKLM (Fig. 4C) or ethanol synthesis (Δ*adhE*, Fig. S2), the only other two pathways that are essential for glycerol fermentation. This directly results from coupling dictated by the PEP dependence of *E. coli* DhaKLM, as overexpression of ATP‐dependent *Cf*DhaKL (which is independent of PEP) in conjunction with GldA restored glycerol consumption in this background whereas the overexpression of PEP‐dependent DhaKLM and GldA did not (Fig. 5D). This result also implies that despite the detrimental impact of FP bypass deletion (Fig. 4C), flux through this pathway appears to be insufficient to serve as the PEP‐independent dissimilation pathway under these conditions. Interestingly, overexpression of *Cf*DhaKL and *E. coli* GldA here also resulted in significant levels of 1,2‐PDO production (0.12 ± 0.04 g L^−1^) indicating that decreasing the PEP dependence of glycerol dissimilation increases the availability of DHAP for 1,2‐PDO production and this route becomes significant in enabling redox balance at alkaline conditions as well. Intriguingly, we also found that at an acidic pH (6.5), a condition under which *glpK* deletion had a less pronounced impact on glycerol consumption, the deletion of *ppsA* alone resulted in a significant reduction in glycerol consumption compared with BW25113 that was not observed at alkaline pH (Fig. S1). This further demonstrates that while GlpK‐GlpABC provides a PEP‐independent glycerol dissimilation pathway, its coupling to HYD activity and the associated pH dependence limits its effective operation in the absence of external electron acceptors. Overall, these results provide strong evidence that operation of the PEP‐independent GlpK‐GlpABC pathway and PEP synthetase activity are the only two ways for *E. coli* to compensate for the stoichiometric constraints created by the PEP dependency of GldA‐DhaKLM.

The above PEP limitations may also have implications for the operation of PEP‐carboxylating enzymes. Synthesis of the required 4‐carbon intermediate oxaloacetate (OAA) and associated tricarboxylic acid cycle intermediates (e.g. α‐ketoglutarate) can proceed from either PEP or pyruvate carboxylation (Fig. 5A). PEP carboxylase (Ppc, encoded by *ppc*) is viewed as the primary anaplerotic route with PEP carboxykinase (Pck, encoded by *pckA*) and malic enzymes (encoded by *maeA* and *maeB*) primarily gluconeogenic enzymes (Sauer and Eikmanns, 2005). These roles have been established for aerobic glycerol utilization, with *ppc* an essential gene (Joyce *et al*., 2006; Martinez‐Gomez *et al*., 2012). In contrast, we found that under alkaline fermentative conditions *pckA* deletion had a pronounced impact on cell growth and glycerol consumption while Δ*ppc* had a negligible impact (Fig. 5C). Deletion of *maeA* or *maeB* also did not have a significant impact, further indicating a previously unknown anaplerotic role for Pck under these conditions (Fig. 5C). To confirm this role was specific to fermentative conditions, *ppc* and *pckA* deletion strains were also tested under aerobic conditions. In agreement with previous findings, glycerol utilization was abolished in the Δ*ppc* strain while the Δ*pckA* strain exhibited glycerol consumption levels similar to BW25113 (Fig. S3).

Since the Pck‐catalysed carboxylation of PEP generates ATP via substrate‐level phosphorylation, this enzyme provides the cells with the ability to increase ATP yield during glycerol fermentation. In addition, Pck has a much higher affinity for PEP (*K*_m_ = 0.07 mM; Krebs and Bridger, 1980) than Ppc (*K*_m_ values ranging from 25 mM to 0.19 mM in the absence and presence of activators, respectively; Izui *et al*., 1983). The *K*_m_ of Pck towards PEP is also very similar to that of DhaKLM (*K*_m_ for PEP of 0.045 mM; Gutknecht *et al*., 2001), and hence, it is able to compete with this essential glycerol dissimilation pathway. Finally, while Pck is subject to allosteric inhibition by PEP (*K*_i_ = 0.06 mM) and ATP (*K*_i_ = 0.04 mM; Krebs and Bridger, 1980), both of these inhibitors are expected to be present at low concentrations during glycerol fermentation.

The affinity of Ppc (*K*_m_ = 0.10 mM; Kai *et al*., 1999) and PckA (*K*_m_ = 13 mM; Krebs and Bridger, 1980) for bicarbonate may also play a role in our findings, especially considering the closed fermentation system employed for anaerobic conditions where the initial and evolved CO_2_ accumulation may enable Pck to overcome its much lower affinity compared with Ppc. The fourfold increase in formate concentration seen in Δ*hycE* and Δ*fdhF* strains implies significant amounts of CO_2_ evolution during fermentations with an intact FHL complex.

### Formation of 6‐carbon precursor metabolites from 3‐carbon intermediates

**Fig. 6:**
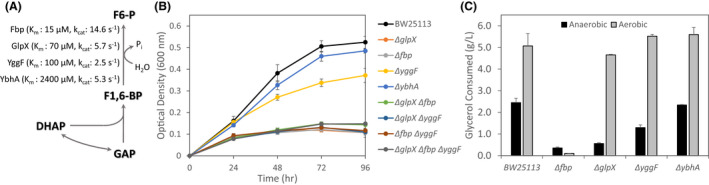
Formation of required 6‐carbon intermediates during glycerol metabolism. (A) Traditional gluconeogenic route for fructose 6‐phosphate (F6‐P) formation from dihydroxyacetone phosphate (DHAP) and glyceraldehyde‐3‐phopshate (GAP) via fructose‐1,6‐bisphosphate (F1,6‐BP). Kinetic parameters for the FBPases Fbp (Kelley‐Loughnane *et al*., 2002), GlpX (Brown *et al*., 2009), YggF (Brown *et al*., 2009) and YbhA (Kuznetsova *et al*., 2006) are shown. (B) *In vivo* cell growth profiles for BW25113 derivatives with deletions to indicated gene(s) encoding FBPases. Number of independent biological replicates: BW25113, *n* = 45; Δ*glpX*, *n* = 3; Δ*fbp*, *n* = 3; Δ*yggF*, *n* = 6; Δ*ybhA*, *n* = 3; Δ*glpX* Δ*fbp*, *n* = 3; Δ*glpX* Δ*yggF*, *n* = 3; Δ*fbp* Δ*yggF*, *n* = 3; and Δ*glpX* Δ*fbp* Δ*yggF*, *n* = 3. (C) Glycerol consumption by BW25113 and individual FBPase deletion strains under anaerobic (96 h) or aerobic (24 h) conditions. Number of independent biological replicates: Anaerobic, same as Panel (B); Aerobic BW2113, *n* = 18; Δ*glpX*, *n* = 3; Δ*fbp*, *n* = 3; Δ*yggF*, *n* = 3; and Δ*ybhA*, *n* = 3.

Generation of 6‐C biosynthetic precursors (e.g. F6‐P) from 3‐C intermediates during glycerol metabolism requires the operation of both glycolytic and gluconeogenic pathways. Under respiratory conditions, 6‐C intermediates are generated via condensation of DHAP and glyceraldehyde‐3‐phosphate to form F1,6‐BP (catalysed by fructose‐bisphosphate aldolase) and subsequent conversion to F6‐P via fructose‐1,6‐bisphosphatase (FBPases; Fig. 6A). While *E. coli* encodes multiple FBPases, including GlpX which is part of the *glpFKX* operon, previous reports have demonstrated that only Fbp (encoded by *fbp*) is required during respiratory growth on glycerol (Donahue *et al*., 2000). However, our *in silico* modelling suggests that the FP bypass could negate the requirement for FBPase activity during fermentative glycerol utilization (Fig. 3). Only in the case of complete FP bypass removal was flux to F6‐P via fructose‐bisphosphate aldolase and FBPase predicted (see previous section). Furthermore, even if the FP bypass appears incapable of serving as the PEP‐decoupling pathway in the absence of GlpK‐GlpABC *in vivo*, the impact observed in the Δ*fsaA* Δ*fsaB* strain (Fig. 4C) could be explained by its ability to generate F6‐P (and other biosynthetic precursors derived from F6‐P).

Given this dichotomy, we evaluated the impact of deleting the FBPases encoded by *fbp* and *glpX*, along with *yggF* and *ybhA*, encoding 2 additional enzymes shown to have FBPase activity (Brown *et al*., 2009). As seen in Fig. 6B, not only did *fbp* deletion have a major impact on cell growth, Δ*glpX* and Δ*yggF* genotypes also exhibited lower cell growth than BW25113. We further constructed double and triple mutants combining deletions of *fbp*, *glpX*, and *yggF* and found that regardless of the combination of FBPase deletions, cell growth remained at similarly low levels to those observed with a single *fbp* or *glpX* deletion (Fig. 6B). With previous reports indicating that only Fbp is required for respiratory glycerol utilization, the individual FBPase deletion strains were also tested under aerobic conditions. While as expected the *fbp* deletion abolished glycerol consumption, the Δ*glpX* and Δ*yggF* strains show no significant difference in glycerol consumption compared with BW25113 (Fig. 6C). These results demonstrate that, unlike respiratory conditions, fermentative metabolism of glycerol requires multiple FBPases, with both GlpX and Fbp playing critical roles.

### Revised metabolic model for the fermentative utilization of glycerol in *Escherichia coli*

Under fermentative conditions, redox balance must be maintained by regenerating oxidized electron carriers using internal electron acceptors. Given the high degree of reduction of glycerol (κ = 4.67) compared to biomass (κ = 4.3), fermentative growth on this substrate results in the next generation of reducing equivalents. Therefore, the ability for microorganisms, including *E. coli*, to utilize glycerol in the absence of external electron acceptors has been linked to the production of organic compounds with a degree of reduction higher than glycerol (e.g. 1,3‐PDO, 1.2‐PDO: κ = 5.33), which represents an electron sink and enables redox balance. Here, we demonstrate that *E. coli* possesses an additional means of achieving overall redox balance by reducing protons to H_2_ through reversible hydrogenase(s). This H_2_‐enabled glycerol fermentation mode in *E. coli* is inherently limited by physiological constraints on the reversibility of hydrogenases and hence restricted to alkaline pH. Under acidic conditions, 1,2‐PDO production remains a critical mechanism for achieving redox balance. Furthermore, the H_2_ evolution mode is enabled by coupling of hydrogenases to G3PDH (GlpABC) and hence to glycerol dissimilation through the GlpK‐GlpABC pathway. However, this respiratory pathway is unable to support glycerol utilization under fermentative conditions in the absence of the GldA‐DHAK route. These aspects, combined with additional stoichiometric and ATP generation constraints during fermentative metabolism of glycerol, result in system‐wide responses in the metabolic network of *E. coli* dependent on the mode redox balance is achieved.

**Fig. 7:**
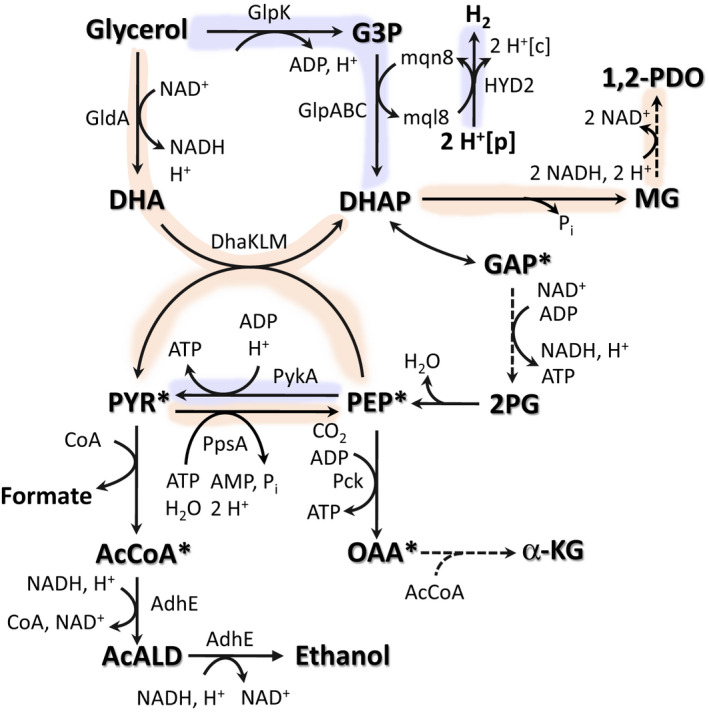
Updated metabolic model for fermentative glycerol metabolism in *E. coli* via complementary 1,2‐PDO‐ or H_2_‐dependent modes operating in a pH‐dependent manner. Under alkaline conditions, H_2_‐dependent mode operates in which HYD‐mediated proton reduction to H_2_ is directly coupled to the menaquinol‐generating GlpK‐GlpABC pathway, which also serves as a PEP‐independent glycerol dissimilation pathway to alleviate stoichiometric constraints resulting from the essential GldA‐DhaKLM pathway (Purple shading). Under acidic conditions, the 1,2‐PDO‐dependent mode exclusively utilizes GldA‐DhaKLM for glycerol dissimilation requiring PEP synthetase to regenerate PEP (Orange shading). Other relevant reactions are represented by the enzyme(s) catalysing each step. Dashed lines illustrate multiple reaction steps. Abbreviations: α‐KG, α‐ketoglutarate; see Fig. 3 for remaining list of abbreviations.

Both the alkaline, H_2_‐enabled or acidic, 1,2‐PDO‐enabled modes of glycerol fermentation require glycerol dissimilation through the NADH‐generating GldA‐DhaKLM route (Fig. 7). From a stoichiometric standpoint, the coupling between glycerol dissimilation and pyruvate synthesis creates a stoichiometric constraint that limits the availability of PEP and other precursor metabolites (e.g. F6‐P, OAA and TCA cycle intermediates). To address these constraints, PEP must be either generated from a different source (e.g. pyruvate) or the PEP dependence decreased. Under alkaline conditions in which hydrogenase reversibility enables a portion of glycerol oxidation to be metabolized via GlpK‐GlpABC (Fig. 7, purple shading), this route serves as a PEP‐independent dissimilation pathway that decouples glycerol utilization from PEP consumption. However, when hydrogenase reversibility is limited, such as under acidic conditions, the use of the ATP intensive PEP synthetase to regenerate PEP is required (Fig. 7, orange shading).

While the use of an NADH‐generating PEP‐independent glycerol dissimilation pathway, such as an ATP‐dependent DHA kinase in conjunction with GldA, alleviates this strain on PEP, despite its potential to natively serve this role the FP bypass in *E. coli* is unable to support glycerol fermentation under the conditions tested here. Combined with the limitations of the GlpK‐GlpABC serving as the PEP‐independent glycerol dissimilation pathway, the strain on PEP and implied low intracellular concentrations has profound system‐wide impacts, including dictating the importance of several enzymes that under fermentative conditions supplement or replace those essential under respiratory conditions. For example, the higher affinity for PEP of Pck compared with Ppc may underlie the importance of the former during fermentative glycerol utilization. Regulatory aspects may also play a role here, as allosteric inhibition of Pck by PEP is likely alleviated. In this context, the tight regulation between glycolysis and gluconeogenesis, specifically the regulatory functions of PEP and F1,6‐BP, also appears to directly contribute to several of our findings. For example, F1,6‐BP activates Ppc (Donahue *et al*., 2000) and notably is a noncompetitive inhibitor of GlpK with high intracellular F1,6‐BP levels potentially decreasing GlpK activity (Thorner and Paulus, 1973). An implied low F1,6‐BP concentration could also contribute, in part, to the lack of a role of Ppc and explain the prominent role of pyruvate kinase II (PykA; Fig. 5C), as pyruvate kinase I (PykF) is strongly activated by F1,6‐BP (Malcovati and Valentini, 1982).

Given its significant regulatory role, the combined action of two FBPases might be required to maintain F1,6‐BP (which is the substrate of FBPases) homeostasis especially as PEP has been proposed as the physiological regulator of Fbp under gluconeogenic growth conditions (Hines *et al*., 2006). Fbp is extremely sensitive to AMP inhibition (50% inhibition by 15 μM AMP; Babul and Guixe, 1983); however, this inhibition is alleviated by PEP. While GlpX and YggF are less catalytically efficient enzymes than Fbp (Fig. 6A), neither have shown such sensitive inhibition by any compound. Thus, while the PEP‐independent glycerol dissimilation pathway utilized under respiratory conditions (GlpK‐GlpD) may help alleviate inhibition of Fbp (due to increased PEP levels), under fermentative conditions with PEP‐dependent glycerol dissimilation and implied low intracellular PEP levels, Fbp may not be fully activated and F1,6‐BP conversion requires a combination of Fbp, GlpX and to a lesser extent YggF.

The requirement to generate ATP and maintain redox balance in the absence of external electron acceptors with a carbon source as reduced as glycerol also provides a rationale for *E. coli* to operate under the H_2_‐enabled mode when conditions permit, despite the challenges of coupling to the GlpK‐GlpABC pathway (i.e. electron donor and pathway regulation). By channelling electrons to hydrogenases, excess reducing equivalents can be consumed through H_2_ evolution, which compared to 1,2‐PDO production: (1) provides a more ATP efficient sink for reducing equivalents, (2) circumvents the requirement of channelling an intermediate upstream of PEP (DHAP) to 1,2‐PDO which further strains PEP availability and (3) enables increased carbon flux to ethanol synthesis further improving ATP yields. This increased ATP yield may underlie our findings that suggest H_2_ production from protons provides a more efficient route to achieve redox balance that facilitates higher growth rates. Additional experiments towards better understanding the conditions required and ability for H_2_ evolution to support glycerol fermentation in *E. coli* can provide new insights on this metabolic process. This may provide a basis for identifying other organisms with this latent capability to extend the range of organisms capable of fermentative glycerol utilization beyond those with the established propanediol dependent models.

## Experimental procedures

### Model implementation, simulation and curation

The genome‐scale model (GEM) iJO1366 for *E. coli*, which includes 1366 genes, 2251 reactions and 1136 unique metabolites, was used as starting point (Orth *et al*., 2011). iJO1366 was further modified to most accurately reflect important enzymes, pathways and mechanisms involved with the current knowledge of fermentative glycerol utilization as described in Appendix S1. External conditions were set to represent minimal medium under anaerobic conditions (no external electron acceptor) as defined elsewhere (Feist *et al*., 2007; Orth *et al*., 2011), and glycerol was set as the only limiting nutrient with an uptake rate value of −20 mmol gCDW^−1^ h^−1^. The non‐growth associated ATP maintenance (ATPM) is considered as 3.15 mmol gCDW^−1^ h^−1^. Flux Balance Analysis (FBA) and Flux Variability Analysis (FVA) were used to predict flux distribution for optimal cell growth and solution spaces, respectively. All simulations were performed using COBRA toolbox v2.0 (Schellenberger *et al*., 2011) in Matlab v7.11.0 (R2010b, The Mathworks, Natick, MA, USA).

### Strains, plasmids and genetic methods

*Escherichia coli* K12 strain BW25113 and single‐gene knockout mutants were obtained from the National BioResource Project (NIG, Mishima, Japan; Baba *et al*., 2006). Strains containing multiple gene deletions were constructed by P1 phage transduction using the single‐gene knockout mutants as donors of specific mutations. All strains are listed in Table S3. All mutations, including single deletion mutants obtained from NIG, were confirmed by polymerase chain reaction using appropriate primers. Plasmids pZSKLMgldA and pZSKLcfgldA used in this study have been previously described (Yazdani and Gonzalez, 2008; Clomburg and Gonzalez, 2011). Molecular biology techniques were performed with standard methods (Miller, 1972; Sambrook *et al*., 2001) or by manufacturer protocol. Strains were kept in 32.5% glycerol stocks at −80°C. Plates were prepared using LB medium containing 1.5% agar with appropriate antibiotics (50 µg ml^−1^ kanamycin, 34 µg ml^−1^ chloramphenicol).

### Culture medium and cultivation conditions

The minimal medium designed by Neidhardt and Bloch (1974) with 1.32 mM Na_2_HPO_4_ in place of K_2_HPO_4_ supplemented with 10 g L^−1^ glycerol and 2 g L^−1^ tryptone (pH 7.2) was used unless otherwise stated. The inclusion of 2 g L^−1^ tryptone has been found to be required for fermentative glycerol utilization but does not compromise the fermentative nature of glycerol utilization (Gonzalez *et al*., 2008; Murarka *et al*., 2008). The redox indicator resazurin (1 mg L^−1^; Ferguson and Cummins, 1978) and reducing agent dithiothreitol (DTT, 1 mM) were included as a means to both detect (resazurin is pink in the presence of oxygen) and eliminate oxygen from the media ensuring strict anaerobic conditions. Chloramphenicol (12.5 µg ml^−1^) and anhydrotetracycline (100 ng ml^−1^) were included for strains transformed with pZS series vectors. Chemicals were obtained from Fisher Scientific (Pittsburgh, PA, USA) and Sigma‐Aldrich (St. Louis, MO, USA).

Anaerobic fermentations were conducted in 17‐ml Hungate tubes (Bellco Glass, Vineland, NJ, USA). Prior to inoculation, oxygen in the medium was removed through the addition of reducing agent (DTT) and storage in an oxygen‐free environment (90% N_2_, 5% CO_2_, 5% H_2_,) provided within a BACTRON I Anaerobic Chamber (Sheldon Manufacturing, Cornelius, OR, USA) until the medium was void of colour. A single colony was used to inoculate 5 ml of media in each tube, which were then incubated at 37°C with rotation in a LabQuake rotator (Fisher Scientific, Pittsburgh, PA, USA) for 96 h in an Isotemp Incubator (Fisher Scientific). Aerobic shake flask experiments were conducted in 25 ml Erlenmeyer flasks (Corning, Corning, NY, USA) using the above defined media without the inclusion of tryptone, resazurin and DTT. Pre‐cultures for these fermentations were prepared by inoculating 5 ml of LB media with a single colony in Hungate tubes and incubating these tubes with rotation at 37°C until an optical density of ~0.4 was reached. An appropriate volume of actively growing pre‐culture was centrifuged, washed twice with minimal media and used to inoculate 5 ml of media in each flask, with a target initial optical density of 0.05. Flasks were capped with a foam plug and incubated at 37°C and 140 r.p.m. in a Lab Companion SI‐600 rotary shaker (Jeiotech, Seoul, Korea) for 24 h.

### Analytical methods and enzyme assays

Optical density was measured at 600 nm using a WPA BioWave CO8000 Cell Density Meter (Biochrom, Cambridge, UK). After centrifugation of the sample, the supernatant was stored at −20°C for high‐performance liquid chromatography (HPLC) analysis. Quantification of glycerol, organic acids, ethanol and other compounds was determined via HPLC using a Shimadzu Prominence SIL 20 system (Shimadzu Scientific Instruments, Columbia, MD, USA) equipped with a refractive index detector and a HPX‐87H organic acid column (Bio‐Rad, Hercules, CA, USA) with operating conditions to optimize peak separation (0.3 ml min^−1^ flowrate, 30 mM H_2_SO_4_ mobile phase, column temperature 42°C). See Appendix S1 for all enzyme assays procedures.

## Supporting information


**Fig. S1**. Glycerol consumption (96 h) of BW25113 and select gene deletions in fermentations with alkaline (7.2) or acidic (6.5) starting pH.
**Fig. S2**. Cell growth, glycerol consumption, and ethanol production (96 h) of BW25113 and alcohol dehydrogenase (Δ*adhE*) strains at alkaline (7.2) starting pH.
**Fig. S3**. Glycerol consumption of BW25113 and PEP carboxylating enzyme (PEP carboxylase, Ppc encoded by *ppc*, and PEP carboxykinase, Pck encoded by *pckA*) deletion strains under anaerobic (96 h) or aerobic (24 h) conditions.
**Table S1**. Flux Variability Analysis (FVA) for the 3 scenarios depicted in Fig. 3.
**Table S2**. Specific activities of glycerol dissimilation in Wild‐Type *E. coli* BW25113 grown under fermentative conditions.
**Table S3**. Strains used in this study.
**Appendix S1**. Supplementary experimental procedures.
