# Supplementary material for: In silico and in vivo analyses reveal key metabolic pathways enabling the fermentative utilization of glycerol in Escherichia coli
Source: Microb Biotechnol. 2021 Oct 26;15(1):289–304. doi: 10.1111/1751-7915.13938 (PMC8719807; doi:10.1111/1751-7915.13938)
Supplement: Supplementary file 1 — Fig. S1. Glycerol consumption (96 h) of BW25113 and select gene deletions in fermentations with alkaline (7.2) or acidic (6.5) starting pH. Fig. S2. Cell growth, glycerol consumption, and ethanol production (96 h) of BW25113 and alcohol dehydrogenase (ΔadhE) strains at alkaline (7.2) starting pH. Fig. S3. Glycerol consumption of BW25113 and PEP carboxylating enzyme (PEP carboxylase, Ppc encoded by ppc, and PEP carboxykinase, Pck encoded by pckA) deletion strains under anaerobic (96 h) or aerobic (24 h) conditions. Table S1. Flux Variability Analysis (FVA) for the 3 scenarios depicted in Fig. 3. Table S2. Specific activities of glycerol dissimilation in Wild‐Type E. coli BW25113 grown under fermentative conditions. Table S3. Strains used in this study. Appendix S1. Supplementary experimental procedures. [file MBT2-15-289-s001.docx]

Supplementary Information

*In silico* and *in vivo* analyses reveal key metabolic pathways enabling the fermentative utilization of glycerol in *Escherichia coli*

James M. Clomburg^1,2,†^, Angela Cintolesi^1,†^, Ramon Gonzalez^1,2^*

^1^Department of Chemical and Biomolecular Engineering Rice University, Houston, TX, USA

^2^ Department of Chemical, Biological, and Materials Engineering, University of South Florida, Tampa, FL, USA

^†^Authors contributed equally to this work

^*^ To whom correspondence should be addressed:

Department of Chemical, Biological, and Materials Engineering, University of South Florida,

ENB 118, 4202 E Fowler Ave, Tampa, FL 33620, USA.

Phone: (813)-974-1223; Email: [ramongonzale@usf.edu](mailto:ramongonzale@usf.edu)

**Supplementary Experimental Procedures**

*Initial model curation.* The *E. coli* GEM iJO1366 (Orth *et al.*, 2011) was modified to most accurately reflect important enzymes, pathways, and mechanisms involved with the current knowledge of fermentative glycerol utilization. Modifications included allowing the formate hydrogen-lyase (FHL) reaction to convert formate into CO_2_ and molecular hydrogen. While this reaction directionality (formation of CO_2_ and H_2_) was considered unfeasible in the original model (Orth et al., 2011; Feist et al., 2007), this enzyme is active under anaerobic conditions and specifically during glycerol fermentation (Murarka et al., 2008; Bagramyan et al., 2002). In addition, the NADP-dependent glycerol-3-phosphate dehydrogenase reaction was considered as reversible in the model, the enzyme responsible (GpsA) is strongly inhibited in vitro by glycerol-3-phosphate (Edgar and Bell, 1978). As flux in the oxidation direction would require the use of glycerol-3-phosphate as the substrate, we limited the reaction directionality to only enable the reduction reaction. Furthermore, based on recent evidence indicating that hydrogenase 2 (HYD2) is a bidirectional enzyme capable of H_2_ oxidation or proton reduction for H_2_ evolution using electrons in the form of menaquinol/demethylmenaquinol (Pinske et al., 2015), the HYD2pp reaction, representing hydrogenase activity coupled to menaquinone in the model, was allowed to operate in a reversable manner rather than the unidirectional activity of H_2_ utilization captured in iJO1366.

Initial adaptation of GEM iJO1366 to simulate the use of glycerol by *E. coli* in minimal medium under anaerobic conditions also indicated operation of pathways that are not functional under the simulated conditions but that enabled redox balance. This included the simultaneous synthesis and degradation of amino acids and nucleotides, futile cycles that have not been reported for wild-type *E. coli* and are not supported by current knowledge (Reitzer, 2005; Jensen et al., 2008). After removing the degradation of amino acids and nucleotides, in silico simulations using FBA predicted the use of sulfate or iron as an electron acceptor through the conversion of sulfate into hydrogen sulfide or oxidizing Fe(III) to Fe(II), respectively. Finally, simultaneous production of spermidine and 5-methylthio-D-ribose were predicted to consume reducing equivalents under certain conditions simulated using FBA, through a pathway that involves production of methionine, an essential amino acid. However, experimental evidence indicates that spermidine is toxic and consequently is not accumulated by E. coli (Limsuwun and Jones, 2000). We therefore restricted the reactions involved exclusively in amino acid degradation, nucleotide degradation and export of amino acids, spermidine, 5-methylthio-D-ribose production, ferrous and hydrogen sulfide to zero flux.

Additional modifications to the overall GEM included the removal of the pyruvate synthase reaction associated with the oxidation of flavodoxin, as experimental evidence associates this reaction with the oxidation of ferredoxin (and not flavodoxin) (Charon et al., 1999), a molecule that is not part of the GEM. In addition, the reaction pyridine nucleotide transhydrogenase (PntAB) that converts NADH into NADPH using a proton exchange pump, was modified to transport only one proton, and not two as included in the original GEM, as this corresponds to a more widely accepted mechanism (Fjellstrom, Johansson and Rydstrom, 1997; Pedersen, Karlsson and Rydstrom, 2008). For simplicity, the sedoheptulose bisphosphate bypass and hexanoate production were eliminated from the model, as they were not of interest in this study.

*Enzyme Assays.* Cells from anaerobic cultures were harvested by centrifugation (10 min, 10,000 × g, 4 °C), washed twice with 9 g/L NaCl and stored as cell pellets at -20 °C. For enzyme assays, cells were resuspended to an optical density of 10 in the buffer used in the specific assay and permeabilized by vortex mixing with chloroform unless otherwise stated (Murarka *et al.*, 2008). Absorbance changes for all assays were monitored in a Biomate 5 spectrophotometer (Thermo Scientific, MA, USA). The linearity of reactions (protein concentration and time) was established for all assays and the nonenzymatic rates were subtracted from the observed initial reaction rates. Enzymatic activities are reported as µmol of substrate per minute per mg of cell protein and represent averages for at least three cell preparations. The activity of glycerol dehydrogenase (Truniger and Boos, 1994), PEP-dependent dihydroxyacteone kinase (Yazdani and Gonzalez, 2008), glycerol kinase (Pettigrew *et al.*, 1998), aerobic and anaerobic glycerol-3-phosphate dehydrogenases were assayed as previously described (Murarka *et al.*, 2008). For the PEP-dependent dihydroxyacteone kinase assay, decryptification buffer (0.1 M-sodium-potassium phosphate, pH 7.2, and 5 mM MgCl_2_) was used for cell washing in place of 9 g/L NaCl mentioned above and cells were disrupted using a toluene-ethanol mixture (1:9 v/v) as previously described (Yazdani and Gonzalez, 2008).

**Supplementary Table 1.** Flux Variability Analysis (FVA) for the 3 scenarios depicted in Figure 3. Values represent the minimum and maximum fluxes through select pathway reactions that support the maximum predicted biomass flux in each scenario.

| **Minimum and maximum flux values of select pathway reactions** | | | | | | |
| --- | --- | --- | --- | --- | --- | --- |
| Reaction/Pathway/Enzyme (*gene*) | Base Case | | ΔHYD | | 20% FPB | |
|  | Min | Max | Min | Max | Min | Max |
| glycerol dehydrogenase  (*gldA*) | 8.67 | 8.67 | 9.99 | 9.99 | 8.67 | 8.67 |
| dihydroxyacetone kinase  (*dhaKLM*) | 0.00 | 8.61 | 0.00 | 8.33 | 6.67 | 8.61 |
| fructose 6-phosphate aldolase  (*fsaA*, *fsaB*) | 0.07 | 8.67 | 1.66 | 9.99 | 0.07 | 2 |
| glycerol kinase  (*glpK*) | 1.33 | 1.33 | 0.01 | 0.01 | 1.33 | 1.33 |
| glycerol-3-P-dehydrogenase  (*glpABC*) | 1.32 | 1.32 | 0.00 | 0.00 | 1.32 | 1.32 |
| 1,2-PDO production | 0.00 | 0.00 | 1.07 | 1.07 | 0.00 | 0.00 |
| Hydrogenase  (*hybOABCDEFG)* | 1.34 | 1.34 | 0.00 | 0.00 | 1.34 | 1.34 |
| triose-phosphate isomerase  (*tpiA*) | 9.92 | 9.92 | 8.87 | 8.87 | 9.92 | 9.92 |
| enolase | 9.69 | 9.69 | 8.69 | 8.69 | 9.69 | 9.69 |
| pyruvate kinase  (*pykA*, *pykF*) | 0.65 | 9.26 | 0.00 | 8.32 | 0.65 | 2.59 |
| pyruvate formate lyase | 9.05 | 9.05 | 8.17 | 8.17 | 9.05 | 9.05 |
| formate hydrogen lyase | 9.04 | 9.04 | 8.16 | 8.16 | 9.04 | 9.04 |
| ethanol production | 8.61 | 8.61 | 7.80 | 7.80 | 8.61 | 8.61 |

**Supplementary Table 2.** Specific activities of glycerol dissimilation in Wild-Type *E. coli* BW25113 grown under fermentative conditions

| Enzyme Assayed (*Gene*) | Activity^a,b^  (µmol/mg protein/min) |
| --- | --- |
| glycerol dehydrogenase (*gldA*) | 0.132 ± 0.008 |
| PEP-dependent dihydroxyacetone kinase (*dhaKLM*) | 0.051 ± 0.002 |
| glycerol kinase (*glpK*) | 0.102 ± 0.008 |
| anaerobic glycerol-3-phosphate dehydrogenase (*glpABC*) | 0.120 ± 0.007 |
| aerobic glycerol-3-phosphate dehydrogenase (*glpD*) | ND^c^ |

^a^All activities measured as described in Materials and Methods and values are reported as average ± standard deviation of triplicate assays

^b^Activities measured in wild-type BW25113 after 72 hours growth

^c^ND: Not detectable (minimum detectable activity was 0.001 µmol/mg protein/min)

**Supplementary Table 3.** Strains used in this study

| Strain | Genotype/Description | Source |
| --- | --- | --- |
| BW25113 | *rrnB3* Δ*lacZ4787* *hsdR514* Δ(*araBAD*)*567* Δ(*rhaBAD*)*568* *rph*-1 | (Baba *et al.*, 2006) |
| BW25113 Δ*”gene*” | BW25113, Δ*”gene” FRT-Kan-FRT* | (Baba *et al.*, 2006) |
| BW25113 Δ*fsaA* Δ*fsaB* | BW25113, Δ*fsaA*::*FRT* Δ*fsaB FRT-Kan-FRT;* sequential deletion of *fsaA* and *fsaB* genes | This Study |
| BW25113 Δ*pykA* Δ*pykF* | BW25113, Δ*pykA*::*FRT* Δ*pykF FRT-Kan-FRT;* sequential deletion of *pykA* and *pykF* genes | This Study |
| BW25113 Δ*glpK* Δ*pps* | BW25113, Δ*glpK*::*FRT* Δ*pps FRT-Kan-FRT;* sequential deletion of *glpK* and *pps* genes | This Study |
| BW25113 Δ*glpX* Δ*fbp* | BW25113, Δ*glpX*::*FRT* Δ*fbp FRT-Kan-FRT;* sequential deletion of *glpX* and *fbp* genes | This Study |
| BW25113 Δ*glpX* Δ*yggF* | BW25113, Δ*glpX*::*FRT* Δ*yggF FRT-Kan-FRT;* sequential deletion of *glpX* and *yggF* genes | This Study |
| BW25113 Δ*fbp* Δ*yggF* | BW25113, Δ*fbp*::*FRT* Δ*yggF FRT-Kan-FRT;* sequential deletion of *fbp* and *yggF* genes | This Study |
| BW25113 Δ*glpX* Δ*fbp* Δ*yggF* | BW25113, Δ*glpX*::*FRT* Δ*fbp*::*FRT* Δ*yggF FRT-Kan-FRT;* sequential deletion of *glpX*, *fbp*, and *yggF* genes | This Study |

**Supplementary Figure 1.** Glycerol consumption (96 hrs) of BW25113 and select gene deletions in fermentations with alkaline (7.2) or acidic (6.5) starting pH. Values shown as a percentage of glycerol consumed by BW25113 at the indicated starting pH. Number of independent biological replicates: BW25113 pH 7.2, n = 45; BW25113 pH 6.5, n = 4; Δ*gldA* pH 7.2, n = 3; Δ*gldA* pH 6.5, n = 2; Δ*dhaK* pH 7.2, n = 3; Δ*dhaK* pH 6.5, n = 2;Δ*glpK* pH 7.2, n = 3; Δ*glpK* pH 6.5, n = 4; Δ*ppsA* pH 7.2, n = 3; Δ*ppsA* pH 6.5, n = 2; Δ*ppsA* Δ*glpK* pH 7.2, n = 6; Δ*ppsA* Δ*glpK* pH 6.5, n = 2.

**Supplementary Figure 2.** Cell growth, glycerol consumption, and ethanol production (96 hrs) of BW25113 and alcohol dehydrogenase (Δ*adhE*) strains at alkaline (7.2) starting pH. Number of independent biological replicates: BW25113, n = 45; Δ*adhE*, n = 3.

**Supplementary Figure 3.** Glycerol consumption of BW25113 and PEP carboxylating enzyme (PEP carboxylase, Ppc encoded by *ppc*, and PEP carboxykinase, Pck encoded by *pckA*) deletion strains under anaerobic (96 hrs) or aerobic (24 hrs) conditions. Number of independent biological replicates: Anaerobic BW25113, n = 45; Aerobic BW2113, n = 18; Anaerobic Δ*ppc*, n = 6; Aerobic Δ*ppc*, n = 3; Anaerobic Δ*pckA*, n = 6; Aerobic Δ*pckA*, n = 3.

**Supplementary References**

Baba, T., Ara, T., Hasegawa, M., Takai, Y., Okumura, Y., Baba, M., Datsenko, K. A., Tomita, M., Wanner, B. L. and Mori, H. (2006) Construction of *Escherichia coli* K-12 in-frame, single-gene knockout mutants: the Keio collection. *Molecular Systems Biology* **2**: 11.

Bagramyan, K., Mnatsakanyan, N., Poladian, A., Vassilian, A. and Trchounian, A. (2002) The roles of hydrogenases 3 and 4, and the F_0_F_1_-ATPase, in H_2_ production by *Escherichia coli* at alkaline and acidic pH. *FEBS Letters* **516(1-3)**: 172-178.

Charon, M. H., Volbeda, A., Chabriere, E., Pieulle, L. and Fontecilla-Camps, J. C. (1999) Structure and electron transfer mechanism of pyruvate: ferredoxin oxidoreductase. *Current Opinion in Structural Biology* **9(6)**: 663-669.

Edgar, J. R. and Bell, R. M. (1978) Biosynthesis in *Escherichia coli* of sn-glycerol 3-phosphate, a precursor of phospholipid - purification and physical characterization of wild-type and feedback-resistant forms of biosynthetic sn-glycerol-3-phosphate dehydrogenase. *Journal of Biological Chemistry* **253(18)**: 3492-7.

Feist, A. M., Henry, C. S., Reed, J. L., Krummenacker, M., Joyce, A. R., Karp, P. D., Broadbelt, L. J., Hatzimanikatis, V. and Palsson, B. O. (2007) A genome-scale metabolic reconstruction for *Escherichia coli* K-12 MG1655 that accounts for 1260 ORFs and thermodynamic information. *Molecular Systems Biology,* 3.

Fjellstrom, O., Johansson, C. and Rydstrom, J. (1997) Structural and catalytic properties of the expressed and purified NAD(H)- and NADP(H)-binding domains of proton-pumping transhydrogenase from *Escherichia coli*. *Biochemistry* **36(38)**: 11331–11341.

Jensen, K. F., Dandanell, G., Hove-Jensen, B. and Willemoes, M. (2008) Chapter 3.6.2: Nucleotides, nucleosides and nucleobases. *Ecosal- Escherichia coli and Salmonella; Cellular and Molecular Biology* Washington, DC: ASM. Available at: [www.ecosal.org](file:///C:\Users\james\Documents\Research\Papers\Glycerol%20GEM%20and%20Experimental\www.ecosal.org).

Limsuwun, K. and Jones, P. G. (2000) Spermidine acetyltransferase is required to prevent spermidine toxicity at low temperatures in *Escherichia coli*. *Journal of Bacteriology* **182(19)**: 5373-5380.

Murarka, A., Dharmadi, Y., Yazdani, S. S. and Gonzalez, R. (2008) Fermentative utilization of glycerol by *Escherichia coli* and its implications for the production of fuels and chemicals. *Applied and Environmental Microbiology* **74(4)**: 1124-1135.

Orth, J. D., Conrad, T. M., Na, J., Lerman, J. A., Nam, H., Feist, A. M. and Palsson, B. O. (2011) A comprehensive genome-scale reconstruction of *Escherichia coli* metabolism-2011. *Molecular Systems Biology* 7.

Pedersen, A., Karlsson, G. B. and Rydstrom, J. (2008) Proton-translocating transhydrogenase: an update of unsolved and controversial issues. *Journal of Bioenergetics and Biomembranes* **40(5)**: 463-73.

Pettigrew, D. W., Smith, G. B., Thomas, K. P. and Dodds, D. C. (1998) Conserved active site aspartates and domain-domain interactions in regulatory properties of the sugar kinase superfamily. *Archives of Biochemistry and Biophysics* **349(2)**: 236-245.

Pinske, C., Jaroschinsky, M., Linek, S., Kelly, C. L., Sargent, F. and Sawers, R. G. (2015) Physiology and Bioenergetics of NiFe -Hydrogenase 2-Catalyzed H_2_-Consuming and H_2_-Producing Reactions in *Escherichia coli*. *Journal of Bacteriology* **197(2)**: 296-306.

Reitzer, L. (2005) Chapter 3.4.7: Catabolism of Amino Acids and Related Compounds. *EcoSal-Escherichia coli and Salmonella: cellular and molecular biology* Washington, DC: ASM Press. Available at: [www.ecosal.org](file:///C:\Users\james\Documents\Research\Papers\Glycerol%20GEM%20and%20Experimental\www.ecosal.org).

Truniger, V. and Boos, W. (1994) Mapping and cloning of *gldA*, the structural gene of the *Escherichia coli* glycerol dehydrogenase. *Journal of Bacteriology* **176(6)**: 1796-1800.

Yazdani, S. S. and Gonzalez, R. (2008) Engineering *Escherichia coli* for the efficient conversion of glycerol to ethanol and co-products. *Metabolic Engineering* **10(6)**: 340-351.
